# A synthetic model simulator for intracranial aneurysm clipping: validation of the UpSurgeOn AneurysmBox

**DOI:** 10.3389/fsurg.2023.1185516

**Published:** 2023-05-31

**Authors:** Razna Ahmed, William Muirhead, Simon C. Williams, Biswajoy Bagchi, Priyankan Datta, Priya Gupta, Carmen Salvadores Fernandez, Jonathan P. Funnell, John G. Hanrahan, Joseph D. Davids, Patrick Grover, Manish K. Tiwari, Mary Murphy, Hani J. Marcus

**Affiliations:** ^1^ Queen Square Institute of Neurology, University College London, London, United Kingdom; ^2^ Wellcome/EPSRC Centre for Interventional and Surgical Sciences (WEISS), University College London, London, United Kingdom; ^3^ Victor Horsley Department of Neurosurgery, National Hospital for Neurology and Neurosurgery, London, United Kingdom; ^4^ Nanoengineered Systems Laboratory, Department of Mechanical Engineering, University College London, London, United Kingdom; ^5^ Institute of Global Health Innovation and Hamlyn Centre for Robotics Surgery, Imperial College London, London, United Kingdom

**Keywords:** aneurysm clipping, education, simulation, validation, surgical simulation and training

## Abstract

**Background and objectives:**

In recent decades, the rise of endovascular management of aneurysms has led to a significant decline in operative training for surgical aneurysm clipping. Simulation has the potential to bridge this gap and benchtop synthetic simulators aim to combine the best of both anatomical realism and haptic feedback. The aim of this study was to validate a synthetic benchtop simulator for aneurysm clipping (AneurysmBox, UpSurgeOn).

**Methods:**

Expert and novice surgeons from multiple neurosurgical centres were asked to clip a terminal internal carotid artery aneurysm using the AneurysmBox. Face and content validity were evaluated using Likert scales by asking experts to complete a post-task questionnaire. Construct validity was evaluated by comparing expert and novice performance using the modified Objective Structured Assessment of Technical Skills (mOSATS), developing a curriculum-derived assessment of Specific Technical Skills (STS), and measuring the forces exerted using a force-sensitive glove.

**Results:**

Ten experts and eighteen novices completed the task. Most experts agreed that the brain looked realistic (8/10), but far fewer agreed that the brain felt realistic (2/10). Half the expert participants (5/10) agreed that the aneurysm clip application task was realistic. When compared to novices, experts had a significantly higher median mOSATS (27 vs. 14.5; *p* < 0.01) and STS score (18 vs. 9; *p* < 0.01); the STS score was strongly correlated with the previously validated mOSATS score (*p* < 0.01). Overall, there was a trend towards experts exerting a lower median force than novices, however, these differences were not statistically significant (3.8 N vs. 4.0 N; *p* = 0.77). Suggested improvements for the model included reduced stiffness and the addition of cerebrospinal fluid (CSF) and arachnoid mater.

**Conclusion:**

At present, the AneurysmBox has equivocal face and content validity, and future versions may benefit from materials that allow for improved haptic feedback. Nonetheless, it has good construct validity, suggesting it is a promising adjunct to training.

## Introduction

Clipping of cerebral aneurysms is a high-risk, technically challenging, and low-volume procedure which presents challenges to training (1). This procedure has significant operative risks, including intraoperative aneurysm rupture, tissue injury, postoperative seizures, and stroke (2). Increasingly, aneurysms are being treated endovascularly using less-invasive interventional techniques further reducing operative exposure and training opportunities for neurosurgical trainees (3). The remaining aneurysms that are unsuitable for coiling are typically complex, further compounding the training challenges. There is a pressing need for a solution to the challenges of training the next generation of specialist neurovascular neurosurgeons (4).

The operating theatre is a challenging environment for skill acquisition and refinement (5). Given limited training opportunities and the complexity of aneurysm clipping, simulation affords trainees a realistic opportunity to practice this procedure in a low-stakes environment without the potential for errors to cause patient harm (6). Surgical simulation has been shown to improve procedural knowledge, technical skills, accuracy, and increase the speed of task completion (7). Deliberate repetition of challenging sections of operative procedures in a controlled environment enables trainees to maximise skill acquisition and refine technique. Indeed, multiple studies have demonstrated that simulation-based deliberate practice is better for technical skill acquisition and skill maintenance when compared to traditional clinical education alone (8). Several accounts in the literature have shown surgical simulation to have translational outcomes (9).

Aneurysm clipping simulators range from physical to virtual reality models, however, often lack arterial vessel pulsatility, intraoperative complications, and inaccurately model the neuroanatomy (6).

Validation studies of simulators are imperative for evaluating the effectiveness of the simulator as a training modality (10). The validity of a simulator includes multiple components, three of which are face validity, content validity, and construct validity (11).

This study aims to assess the validity of a 3D-printed model for cerebral aneurysm clipping for use in simulation. This will evaluate the face, content, and construct validity of the model through multiple metrics.

## Methods

Ethical approval for this study was approved by University College London Research Ethics Committee (17819/001).

### Participants

Twenty-eight surgeons were recruited from multiple centres across the UK. This included both consultant surgeons and trainees. These surgeons were classified into an expert cohort (*n* = 10) and a novice cohort (*n* = 18). Surgeons were classed as experts if they had clipped an aneurysm as first operators and novices otherwise. Verbal consent was obtained prior to inclusion. The sample size was derived from precedence in literature where median sample size is 15 (10–21) with 24% (16%–43%) of total participants being experts (5).

### Model

The AneurysmBox (UpSurgeOn, Milan, Italy) is a benchtop simulator which has been designed to simulate a cerebral aneurysm. The simulator mimics the brain lobes, surrounding vasculature, aneurysms, and cranial nerves. This is developed for manual neurovascular training. The model has been manufactured using 3D-printing technology with silicones and resins. This model is reusable with no moving parts. The model contains several aneurysms through a pterional craniotomy window. The terminal internal carotid artery (ICA) saccular aneurysm was the focus of this study. There is no arachnoid layer, blood flow, and CSF. The model was also supplied with replaceable craniotomy caps which were not used for this study. The model has a smartphone-linked virtual reality (VR) component that illustrates vasculature; however, this overlay was incompatible with the operative microscope.

### Task

**Figure 1:**
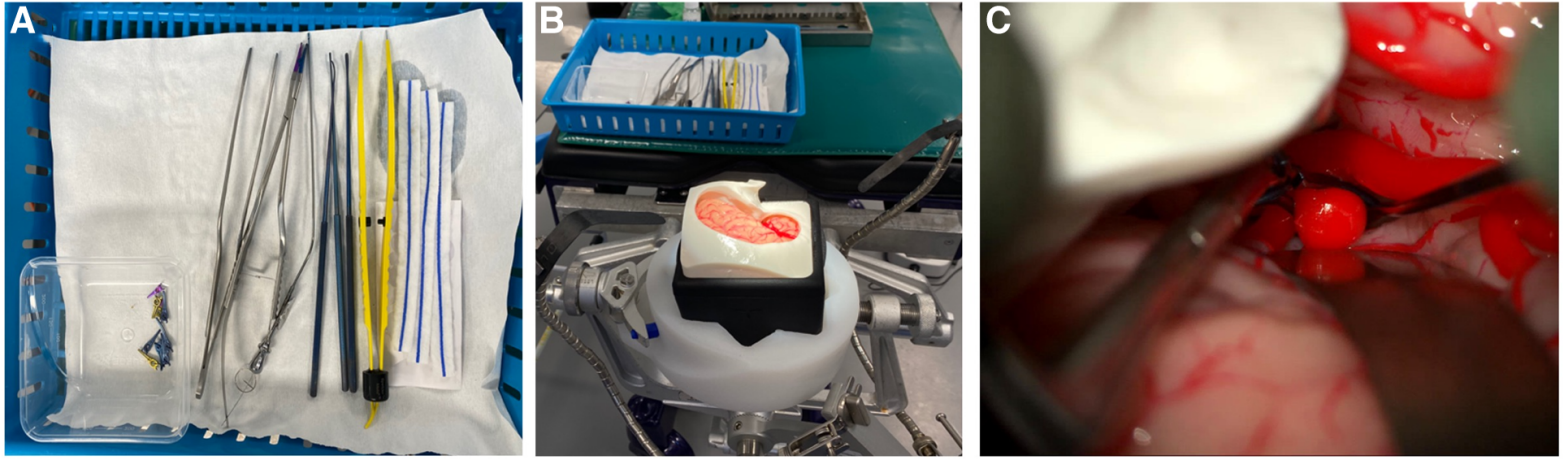
The surgical task (**A**) equipment provided for surgical task (**B**) Set-up for simulation (**C**) operating microscope view of AneurysmBox (UpSurgeOn) with brain lobe retracted to expose aneurysm for clip application.

Participants performed an aneurysm clipping task, exposing, and clipping the terminal ICA aneurysm. Participants were provided with a choice of instruments including retractors, bipolar forceps, Rhoton dissectors, bayonet forceps, suction, Lazic aneurysm clip applicator, and a selection of clips (Figure 1). There was no time limit for the task and each participant completed the task once. Time to task completion was recorded.

### Outcomes

To assess face and content validity, each expert participant was asked to complete a post-task questionnaire to evaluate the simulator (Supplementary Appendix S1). Questions were formatted using a five-point Likert scale. Section 1 of the questionnaire related to the face validity and section 2 related to the content validity of the model. Only experts were asked to complete these sections as is standard in the face and content validation. The questionnaire was adapted from FJ Joseph et al., 2020 (12). All participants were asked for qualitative feedback.

To assess construct validity, we compared multiple metrics including the modified Objective Structured Assessment of Technical Skills (mOSATS) and Specific Technical Skills (STS) (Supplementary Appendix S2). In both cases, videos of each participant performing the task were recorded using a ZEISS Kinevo Operative Microscope (Carl Zeiss Co, Oberkochen, Germany), trimmed to the minute just before clip application, and then scored by five independent blinded neurosurgeons. The mOSATS scale is used to assess the technical skills of surgical trainees, originally validated by Niitsu et al. (13). Non-relevant aspects of the scale were removed leaving six domains. Each domain is scored out of 5, giving a total score of 30. A separate task-specific scale, STS, was derived from Intercollegiate Surgical Curriculum Programme (ISCP) procedure-based assessment for aneurysm clipping, literature review and consultation with expert authors. This scale contains four procedure-specific domains. These domains are scored out of 5, giving a total score of 20. The interrater reliability (IRR) between assessors was calculated using the intraclass correlation coefficient (ICC) to measure internal reliability.

Alongside these video metrics, we also compared forces exerted during the task. All participants were required to wear force-sensitive gloves as previously reported by Layard Horsfall et al. (14) These gloves allow the measurement of the force applied by the dominant thumb to the instrument, and were calibrated to indirectly measure the force applied to brain tissue using the instrument.

### Statistical analysis

Likert data was analysed by assigning each rank a value and calculating the median score. Median mOSATS and STS scores were analysed for differences between experts and novices using the Mann–Whitney *U* test where a *p*-value of <0.05 was deemed to be statistically significant.

The IRR between assessors was calculated using ICC, where ICC > 0.8 suggests a high IRR. Internal consistency is measured using Cronbach *α*, where *α* > 0.80 is considered good. Spearman’s rho was used to determine the concurrent validity of the newly devised STS score relative to mOSATS, where rho = 1 is perfect correlation. Force data is presented as median ± IQR.

Data were analysed using StataMP, Version 17.0 (StataCorp, Texas, USA) and SPSS, Version 26.0 (IBM, N.Y., USA).

## Results

### Demographics

Ten expert and eighteen novice surgeons completed the task (Table 1). The expert surgeons had clipped a median of 60 aneurysms (IQR 15–100) aneurysms independently. The novice surgeons had observed a median of 3.5 (IQR 0–7) aneurysm clipping procedures.

**Table 1:** Participant demographics.

Skill	n (Number of participants)	Handedness (Right: Left)	Years of neurosurgical training^a^	Estimated aneurysms operated independently^a^
Experts	10	9:01	13.5 (11-20.5)	60 (15-100)
Novices	18	18:00	0.42 (0.25-1.83)	0 (0–0)

^a^ Median (Lower Quartile—Upper Quartile).

### Face and content validity

**Figure 2:**
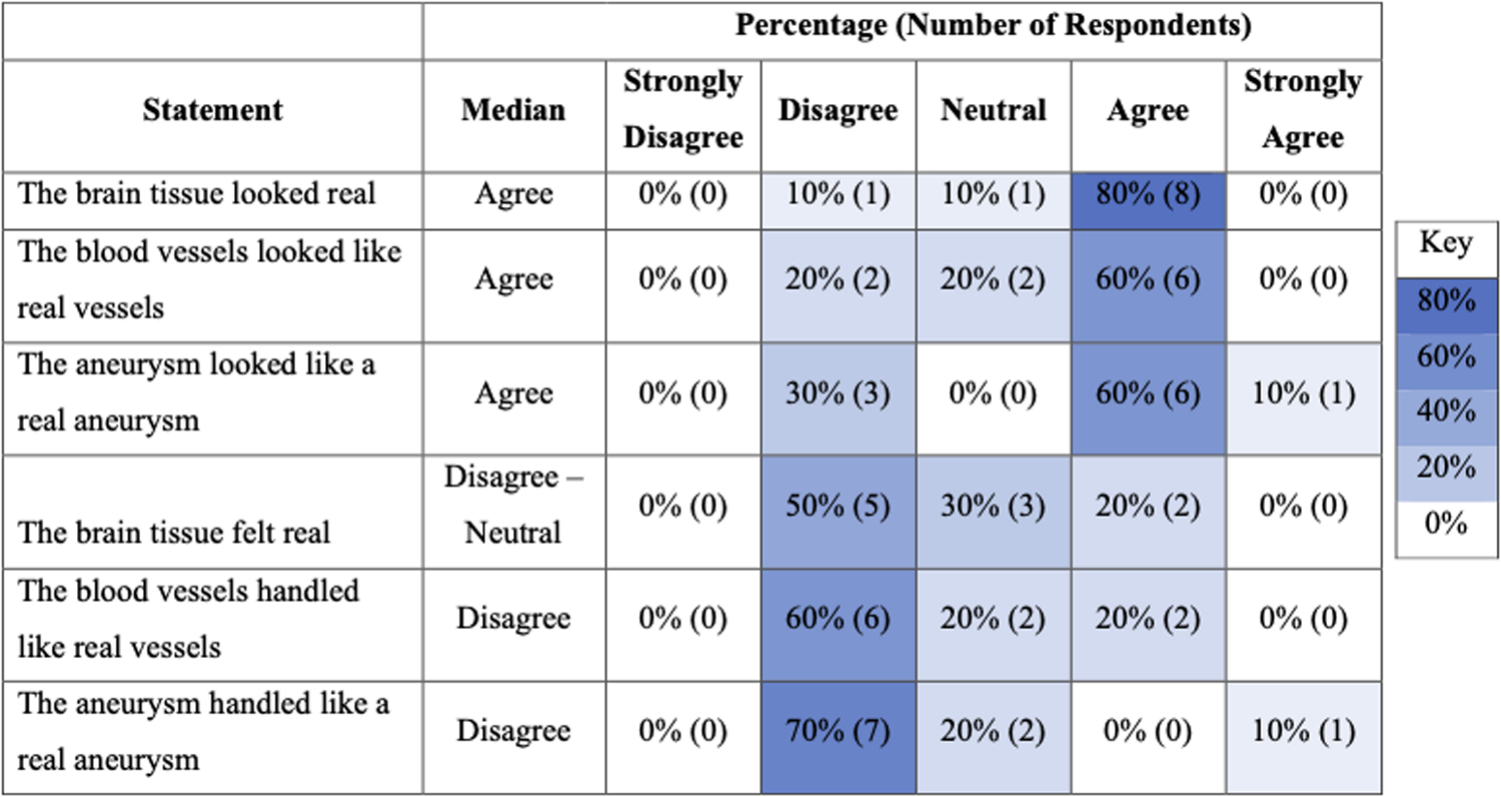
Heat map illustrating distribution of responses for face validity of model.

**Figure 3:**
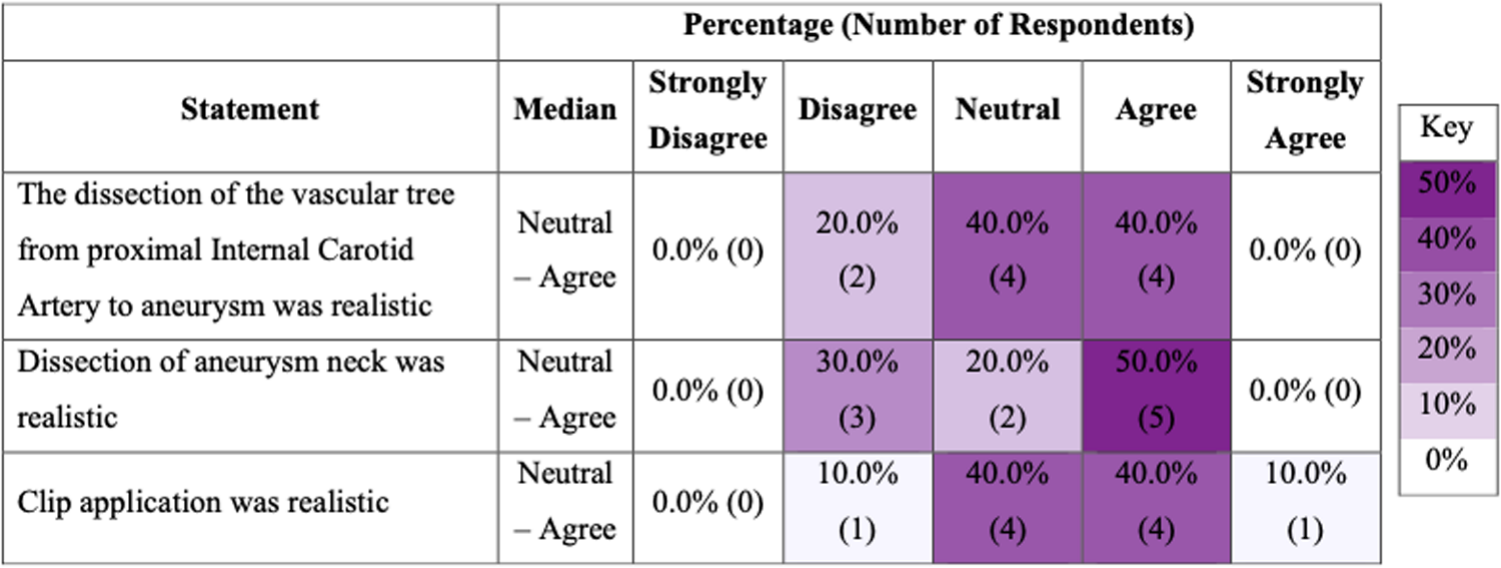
Heat map illustrating distribution of responses for content validity of model.

Ten experts completed post-task questionnaires assessing face and content validity. Eight experts (8/10) agreed that the brain tissue looked realistic but only two (2/10) agreed that it felt realistic (Figure 2). Five experts (5/10) agreed that the dissection of the aneurysm neck was realistic, and the same number (5/10) agreed that the clip application was realistic (Figure 3).

### Construct validity

The median time taken by experts was 3.5 min (1.8–4.5) and for novices was 3.0 min (2.3–4.1).

**Figure 4:**
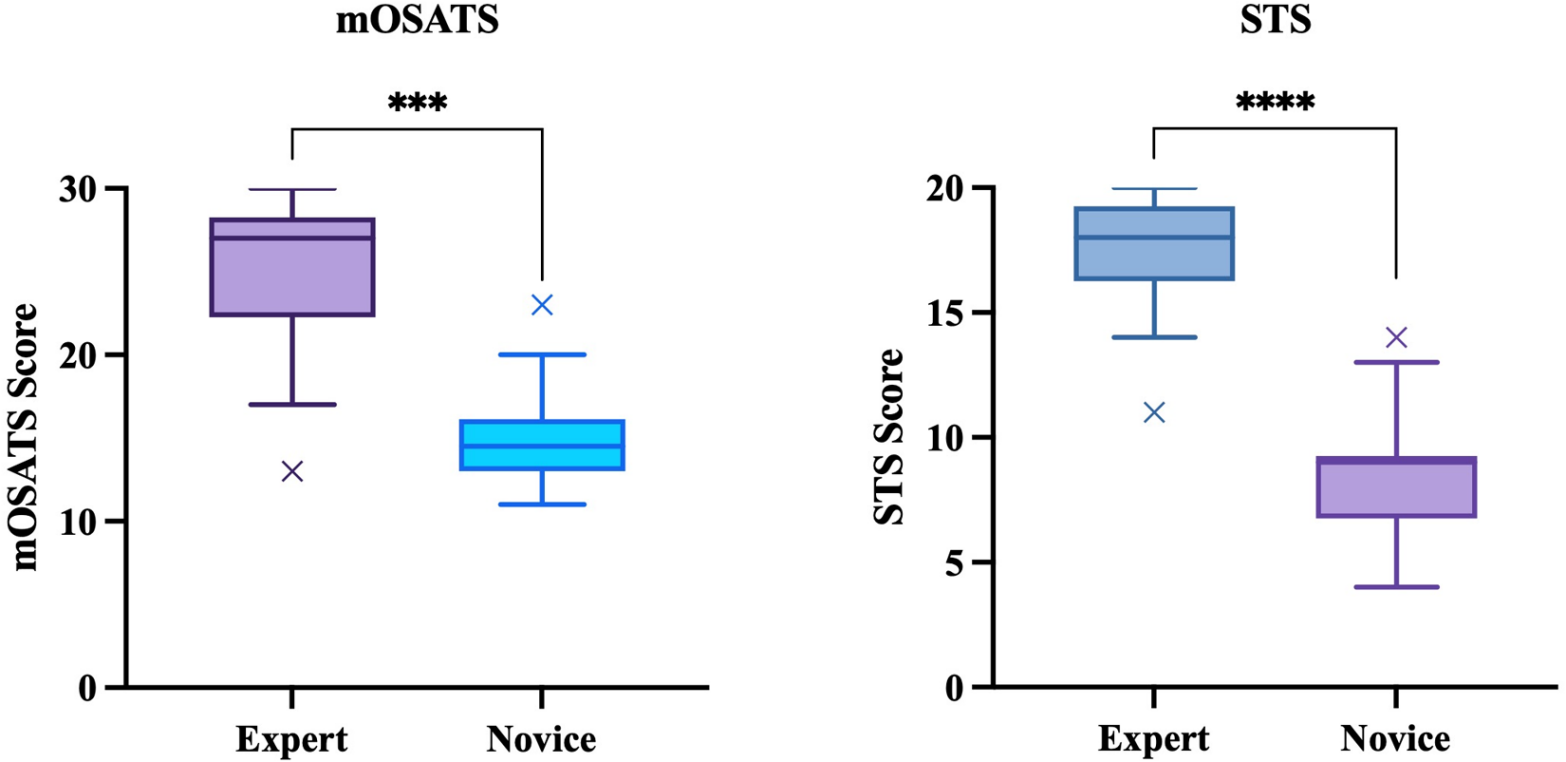
Construct validity: mOSATS and STS scores of experts and novices. Boxplots represent the median (solid line), interquartile range (box margins), minimum and maximum (whiskers), and outliers (x). Outliers are defined as values outside of LQ/UQ ± 1.5xIQR. ***/****p < 0.0001, Mann–Whitney *U*.

The median STS score for experts was 18 (17.25–19) and 9 (7–9) for novices (*p* < 0.01, Mann–Whitney *U*). The median mOSATS score for experts was 27 (24.25–27) and 14.5 (13–15.375) for novices (*p* < 0.01, Mann–Whitney *U*) (Figure 4). There was a significantly high IRR for both STS (*ICC* = 0.95) and mOSATS (*ICC* = 0.93). There was a strong positive correlation between the STS and mOSATS scores (*rho* = 0.835, *p* < 0.01), indicating good concurrent validity. Internal consistency was analysed using Cronbach *α*, where *α* = 0.938, indicating good reliability. All individual rater scores demonstrate excellent consistency (Table 2). Deletion of any single item did not result in a higher *α* score (Table 3).

**Table 2:** Individual rater Cronbach *α* score.

Rater	1	2	3	4	5	Overall
Cronbach *α*	0.911	0.974	0.930	0.919	0.935	0.938

**Table 3:** Interrater agreement and Cronbach *α* for individual scoring dimensions of specific technical skills.

Scoring dimension	Cronbach *α* scale, if deleted	Interrater reliability, ICC (95% CI)
Item 1: Appropriate Exposure	0.910	0.916 (0.854–0.957)
Item 2: Safe retraction	0.929	0.877 (0.787–0.936)
Item 3: Correct clipping	0.929	0.938 (0.892–0.968)
Item 4: Identify branching	0.905	0.936 (0.890–0.967)

ICC, Intraclass Correlation Coefficient; CI, Confidence Interval.

**Figure 5:**
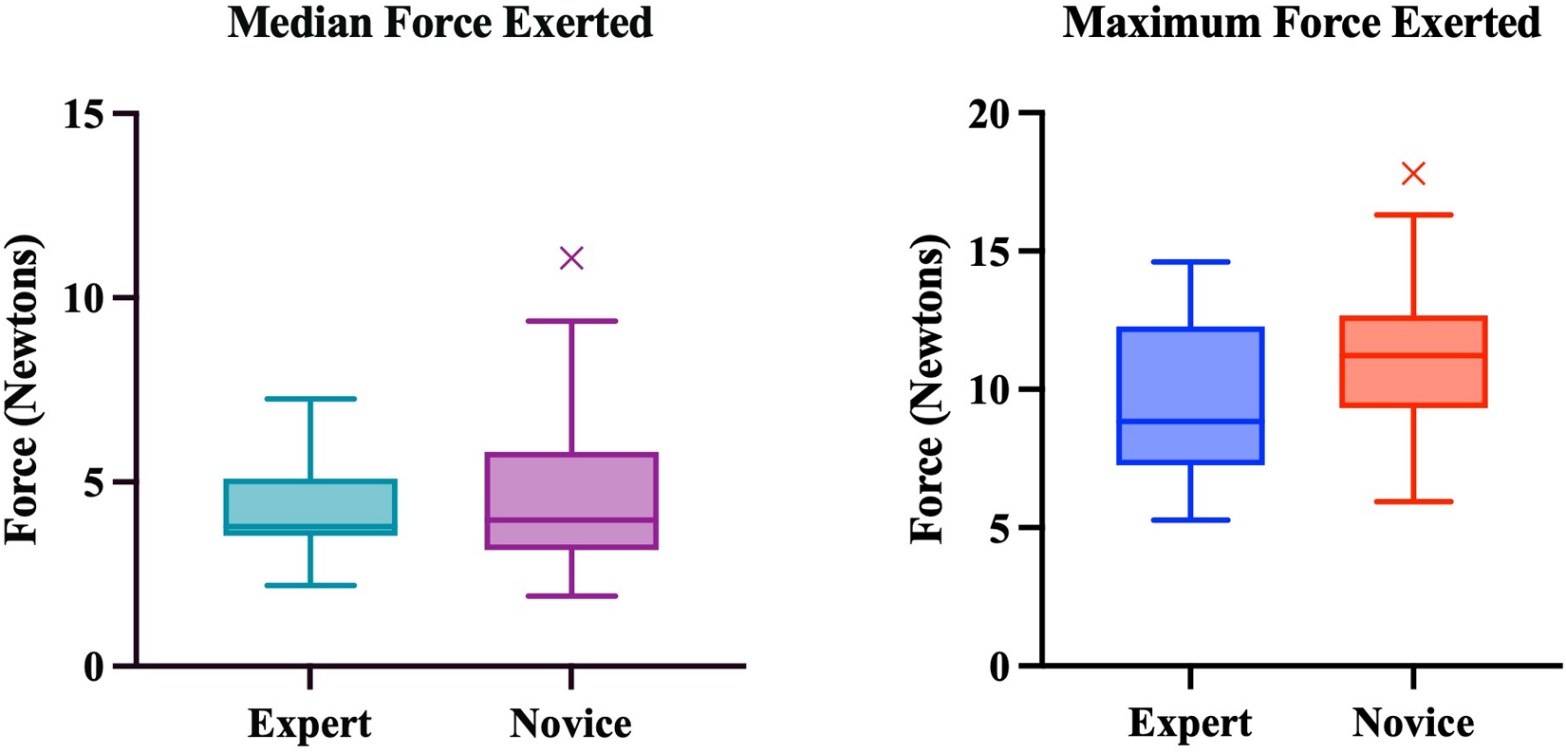
Median and maximum forces exerted by experts and novices. Boxplots represent the median (solid line), interquartile range (box margins), minimum and maximum (whiskers), and outliers (x). Outliers are defined as values outside of LQ/UQ ± 1.5xIQR. No statistically significant difference was noted between expert and novice groups for both Median (*p* = 0.774) and Maximum (*p* = 0.137) Forces.

Overall, there was a trend towards experts exerting less force, however, these differences were not statistically significant (Figure 5).

### Qualitative feedback

All participants felt the model was anatomically realistic and helped with visuospatial representation. Key drawbacks which experts commented on included the stiffness of the material (*n* = 6), lack of arachnoid (*n* = 6), and lack of cerebrospinal fluid (CSF) (*n* = 3).

## Discussion

### Principal findings

To the author's knowledge, this is the first study to evaluate the face, content, and construct validity of the AneurysmBox. The AneurysmBox demonstrated construct validity, but face and content validity scores were equivocal.

Face validity or realism encompasses both anatomical appearance and haptic feedback. Experts agreed the AneurysmBox looked visually realistic. However, there was strong disapproval regarding the haptics. This is likely because the materials made the model difficult to manipulate. The model tissue had lower compliance than real tissue leading to greater forces required for retraction. The blood vessels were also difficult to collapse. Previous studies validating synthetic models have commented on haptic realism limiting face validity. Experts also commented on the absence of arachnoid hindering the realism of the model, however, this is yet to be successfully replicated well in synthetic models.

Content validity is the suitability of the model for anatomical and procedural training purposes. Expert opinions were indeterminate regarding the content validity of the model and whether it improved procedural teaching. This is likely due to the materials used and the absence of key components such as arachnoid, pulsation of blood vessels, and bleeding from vessels.

Construct validity, the ability of the model to delineate between expert and novice surgeons was evaluated by comparing differences between performance scores (STS and mOSATS). The model was able to differentiate between expert and novice surgeons to a high degree, evidenced by significant differences between mOSATS and STS. Interestingly, the STS scale was more sensitive in distinguishing between experts and novices and had less variation amongst results in each group. This can be attributed to the stark contrast in procedural knowledge between groups, as novice surgeons have limited exposure to vascular neurosurgery limiting their knowledge of the procedure. The mOSATS was less sensitive to variations in novice performance, likely because most novice surgeons had some surgical experience and therefore obtained a good global rating.

Previous studies have shown that experts exert less force during all steps of an operation (15). Our findings showed that in general, the expert cohort exerted less force throughout however, these findings were not statistically significant. This is likely a limitation of the model as previous studies indicate that experts exert less force (16). In theory, the difference in forces exerted between the groups should be more distinct however, this can be accredited to model stiffness which required greater force for retraction.

This study also demonstrates that the STS scale is a valid scoring system to assess procedure-specific knowledge of aneurysm clipping. STS scores strongly correlated with mOSATS and were able to delineate between expert and novice participants more accurately than mOSATS alone. There was also a high degree of internal consistency within the sample.

### Comparison with other studies

Synthetic models allow for a haptic response, 3D spatial orientation, and operative microscope practice (17). At the time of writing, six other aneurysm clipping synthetic models have been previously evaluated (12, 17–21). All these studies assessed simulator realism and suitability for training using a post-task questionnaire and concluded that the simulators had good anatomical representation and mimicked the operative procedure well. Previous studies using synthetic models have also commented on haptics limiting realism (18, 21, 22). The absence of arachnoid is another limitation (12, 18–21). Only one study assessed the construct validity, which is a key limitation of many validation studies (23).

Joseph et al. (12) evaluated construct validity by comparing clipping performance between expert and novice surgeons. Their study showed that 44.5% (*n* = 4) of experts successfully clipped the aneurysm compared to 6.3% (*n* = 1) of novices. Belykh et al. (4) assessed the construct validity of a placenta-based aneurysm clipping model with an Objective Structured Assessment of Aneurysm Clipping Skills (OSAACS) scale which contained elements of both OSATS and task-specific items, with elements very similar to our STS scale. However, here the scoring occurred during the simulation with an unblinded proctor (4).

Using force data as a performance metric is a novel methodology that has not been previously described. Our findings were in line with previous findings that experts exert less force however, the significance of these differences was mitigated by model stiffness. Marcus et al. (15) analyzed force data to evaluate the construct validity of a “smart” force-limiting instrument. It is difficult to interpret our findings in context and compare them to real-time retraction forces due to the nature of the simulation.

The AneurysmBox lacked some key elements of the operative procedure taking away from the realism, such as lack of vessel pulsatility, and aneurysm rupture (6). Other synthetic model simulators have been able to replicate pressurized arterial blood flow, pulsatility, and bleeding using red dye, all of which improved the realism (12, 19). Some features, such as dura, have not been effectively replicated yet by any benchtop synthetic model, thus leaving great room for model improvement (23).

While novice trainees may find it helpful to use a model of this sort for familiarization with the anatomy and approach, patient-specific synthetic models have gained popularity among advanced trainees and experts, particularly in challenging cases where endovascular treatment is unsuitable. These models, often created with 3D printing technology, allow for preoperative planning and simulation, facilitating a more personalized and precise approach to treatment (22). There may be a role for combining the AneurysmBox with patient-specific components in the future.

The cost-effectiveness and reusability of the AneurysmBox make it an attractive alternative to traditional training adjuncts such as cadavers, particularly for those early in their training. Compared to cadavers, the AneurysmBox is considerably more affordable, with a price of £500, and discounts available for larger orders (24). Additionally, the model’s reusability and environmental advantages provide further benefits, making it a more practical training solution, especially lower-income countries where resources are limited (25).

### Strengths and limitations

To the author's knowledge, this is the first study to evaluate the face, content, and construct validity of the AneurysmBox. The sample size used is much higher than previous benchtop simulator validation studies with a significantly large proportion of experts.

This study rigorously evaluated the construct validity of the simulator by comparing various performance metrics between expert and novice surgeons, this is often missed in previous studies (23). The surgical task closely matched the intended task of the simulator. The STS and mOSATS scores allowed for an objective assessment of participant performance; together these scores provided a holistic view of procedural-specific knowledge and general surgical skills. The blinding of proctors to participants and using five proctors to evaluate the participant's performance significantly reduces bias. There was a high degree of IRR, evidenced by a significant ICC, demonstrating strong internal validity. This study also included a relatively large number of expert participants compared to other validation studies.

To maximize the likelihood of finding construct validity, we included the novice participants with an average of 0.42 years of training, compared to expert participants with an average of 13.5 years of experience. The inclusion of an intermediate group would strengthened our findings of construct validity further.

A key limitation of this study is that only 1-minute videos prior to clip application were used for scoring, so participant skills during other phases of the task were not evaluated. Both STS and mOSATS scores were subject to rater bias, however, multiple raters were used to mitigate this risk. The concurrent validity of the model was not evaluated in this study; this looks at the translation of skills learnt in simulation into the operative theatre. This cannot be done due to pragmatic constraints and patient safety concerns (26).

This study used a traditional validation framework (face, content, and construct) to demonstrate the validity of the simulator instead of employing contemporary frameworks such as the Messick framework, albeit there is considerable overlap. The main reason for this is that less than 10% of validation studies employ the Messick framework and there is little evidence to show that this supersedes more conventional validation strategies. Using a traditional framework also makes this study more comparable to previously published literature.

This model also has a VR component which allows for it to be used as a hybrid simulator. This additional component was not used in this study however, future research should investigate whether VR combined with a physical model is superior to using VR or synthetic models alone as a training modality.

Future studies analysing the learning curve through repeated simulator use by experts and novices can demonstrate if simulation leads to improvement in task performance and its supplementary metrics.

## Conclusion

At present, the AneurysmBox has equivocal face and content validity, and future versions may benefit from materials that allow for improved haptic feedback. Nonetheless, it has good construct validity, suggesting it is a promising adjunct to training.

Future studies should look at evaluating the predictive validity of the AneurysmBox to determine whether it has translational outcomes.

## Data Availability

The raw data supporting the conclusions of this article will be made available by the authors, without undue reservation.
